# Interoceptive awareness in a clinical setting: the need to bring interoceptive perspectives into clinical evaluation

**DOI:** 10.3389/fpsyg.2024.1244701

**Published:** 2024-06-12

**Authors:** Paola Solano Durán, Juan-Pablo Morales, David Huepe

**Affiliations:** ^1^Center for Social and Cognitive Neuroscience (CSCN), School of Psychology, Universidad Adolfo Ibáñez, Santiago de Chile, Chile; ^2^Oficina de Equidad de Género, Instituto Tecnológico de Costa Rica, Cartago, Costa Rica; ^3^Facultad de Educación Psicología y Familia, Universidad Finis Terrae, Santiago, Chile; ^4^University of Sydney Business School, Darlington, NSW, Australia

**Keywords:** interoceptive awareness, mental health, MAIA, embodiment, interoception

## Abstract

Interoceptive awareness (IA) is crucial to understanding mental health. The Multidimensional Assessment of Interoceptive Awareness (MAIA) scale, available in approximately 30 languages, has gained global recognition for its research applicability. This review highlights the critical importance of integrating IA evaluation in clinical settings, advocating for the MAIA scale’s potential as a screening tool. Through an examination of academic databases, including Scopus, PubMed, Google Scholar, and J-STOR, our analysis spans seven mental health domains: eating disorders (ED), depression, stress, anxiety, autism spectrum disorder (ASD), chronic pain, and suicide ideation (SI). Thirty-eight studies showed links between several dimensions of IA with different disorders. That is, ED was related to Body Trust and Self-Regulation; anxiety to Body Listening, Emotional Awareness, and Self-Regulation; depression to Noticing and Emotional Awareness; ASD to Trusting, Emotional Awareness, and Noticing; chronic pain to Not-Worrying and Self-Regulation; and SI with Trusting. These insights hold profound implications for both clinical practice and mental health research. Integrating IA assessments into standard clinical protocols has the potential to improve our understanding of pathology, enrich patient care, and enhance therapeutic strategies.

## Introduction

The dynamic interplay between the body and brain forms a complex nexus, significantly influencing our cognition, emotions, and overall mental health (Nord and Garfinkel, 2022). This intricate dialogue is mediated by signals within our bodies, providing crucial information about our physical and psychological states. These signals undergo a sensing, encoding, and interpretation process known as interoceptive awareness (IA) (Matiz et al., 2020). IA encompasses the nuanced processing of internal bodily cues, enabling the detection, understanding, and integration of signals from the body’s interior landscape (Nord and Garfinkel, 2022). This vital process is pervasive across all major biological systems, playing a key role in maintaining homeostasis and allostatic balance within the body (Kleckner et al., 2017).

Interoception may occur below the level of consciousness. Typically, signals from the body reach the level of consciousness because homeostasis is out of balance (e.g., when breathing or heart rate increase due to panic attacks, anxiety, and stress) (Berntson and Khalsa, 2021). However, interoception extends beyond these immediate physiological responses. It is deeply interconnected with impulsive processes, affective states, motivations, adaptive reactions, cognition, and emotional experiences. These aspects play pivotal roles in maintaining homeostatic balance, regulating bodily functions, and ensuring survival (Khalsa et al., 2018). IA might serve as a distinctive marker for potentially harmful mental phenomena such as intrusive thoughts or ruminations. This hypothesis underscores the necessity of incorporating IA into clinical assessments, suggesting its potential as a critical factor in identifying and understanding mental health conditions.

### Evaluating IA: the role of the MAIA instrument

Various methods to assess AI can be broadly categorized into interoceptive accuracy or sensitivity—evaluated through objective and behavioral measures—and interoceptive sensibility or awareness, gauged *via* self-report instruments and questionnaires. This delineation underscores the importance of recognizing and attending to bodily sensations across different sensory modalities (Vig et al., 2021). We focus on the latter, specifically utilizing the Multidimensional Assessment of Interoceptive Awareness (MAIA) tool.

Developed by Mehling et al. (2012), the MAIA is a comprehensive self-report scale designed to assess adult IA. It evaluates body awareness, reflecting sensory perceptions from bodily states, actions, and processes, alongside subjective appraisals—attitudes, beliefs, and experiences contextualized within sociocultural backgrounds (Mehling et al., 2012). The utility of the MAIA spans experimental research and mind–body therapies, offering insights into aspects of personal experience (Shoji et al., 2018; Fiskum et al., 2023).

The MAIA highlights the potential use of IA in clinical diagnosis and therapy for various mental conditions, as evidenced by its sensitivity to changes in body awareness and attentional styles (Mehling et al., 2012, 2018). Scores on the MAIA indicate adaptability, whereas higher scores suggest a mindful approach to interoceptive cues (Mehling et al., 2018; Mul et al., 2018; Flasinski et al., 2020; Yang et al., 2021; Yang H. X. et al., 2022). The scale features 32 items across eight subscales (Table 1), with recent additions highlighted in the updated MAIA 2 version (Table 1). Available in approximately 30 languages, the MAIA facilitates global research accessibility through the Osher Center for Integrative Medicine website,^1^ accessible without charge.

**Table 1:** Dimensions, subscales, and items of MAIA and MAIA 2.

**Dimension**	**Subscale**	**Items**
(1) Awareness of Body Sensations	Noticing: Awareness of uncomfortable, comfortable, and neutral body sensations	1. When I am tense, I notice where the tension is located in my body.
		2. I notice when I am uncomfortable in my body.
		3. I notice where in my body I am comfortable.
		4. I notice changes in my breathing, such as whether it slows down or speeds up.
(2) Emotional Reaction and Attentional Response to Sensations	Not-Distracting: Tendency to ignore or distract oneself from sensations of pain or discomfort	5. I do not notice (I ignore) physical tension or discomfort until they become more severe.
		6. I distract myself from sensations of discomfort.
		7. When I feel pain or discomfort, I try to power through it.
		**8. I try to ignore pain**
		**9. I push feelings of discomfort away by focusing on something**
		**10. When I feel unpleasant body sensations, I occupy myself with something else so I do not have to feel them.**
	Not-Worrying: Emotional distress or worry with sensations of pain or discomfort (reversed)	11. When I feel physical pain, I become upset.
		12. I start to worry that something is wrong if I feel any discomfort.
		13. I can notice an unpleasant body sensation without worrying about it.
		**14. I can stay calm and not worry when I have feelings of discomfort or pain.**
		**15. When I am in discomfort or pain I cannot get it out of my mind**
(3) Capacity to Regulate Attention: ability to stay focused when facing numerous sensory stimuli competing for attention	Attention Regulation: Ability to sustain and control attention to body sensation	16. I can pay attention to my breath without being distracted by things happening around me.
		17. I can maintain awareness of my inner bodily sensations even when there is a lot going on around me.
		18. When I am in conversation with someone, I can pay attention to my posture.
		19. I can return awareness to my body if I am distracted.
		20. I can refocus my attention from thinking to sensing my body.
		21. I can maintain awareness of my whole body even when a part of me is in pain or discomfort.
		22. I am able to consciously focus on my body as a whole.
(4) Awareness of Mind–Body Integration: access to more developed levels of body awareness	Emotional Awareness: Awareness of the connection between body sensations and emotional states	23. I notice how my body changes when I am angry.
		24. When something is wrong in my life I can feel it in my body.
		25. I notice that my body feels different after a peaceful experience.
		26. I notice that my breathing becomes free and easy when I feel comfortable.
		27. I notice how my body changes when I feel happy/joyful.
	Self-Regulation: Ability to regulate psychological distress by attention to body sensations	28. When I feel overwhelmed, I can find a calm place inside.
		29. When I bring awareness to my body, I feel a sense of calm.
		30. I can use my breath to reduce tension.
		31. When I am caught up in thoughts, I can calm my mind by focusing on my body/breathing.
	Body Listening: Actively listen to the body for insight	32. I listen for information from my body about my emotional state.
		33. When I am upset, I take time to explore how my body feels.
		34. I listen to my body to inform me about what to do
(5) Trusting Body Sensations	Trusting: Experiences one’s body as safe and trustworthy	35. I am at home in my body.
		36. I feel my body is a safe place.
		37. I trust my body sensations.

Words in bold are the new items and correspond to MAIA 2.

However, the reliability and dimensionality of the instrument have been substantiated. Reis (2019) employed a range of structural equation modeling techniques, including maximum likelihood confirmatory factor analysis (ML-CFA), exploratory structural equation modeling (ESEM), and Bayesian structural equation modeling (BSEM). The results varied from poor to excellent fit, highlighting the challenges in accurately measuring IA (Reis, 2019). Furthermore, the Rasch Measurement Theory confirmed the scale’s reliability, though it necessitated the exclusion of three items due to insufficient factor loading. These items were item 5, “I do not notice physical tension or discomfort until they become more severe”; item 16, “I can maintain awareness of my whole body even when a part of me is in pain or discomfort”; and item 23, “When I feel overwhelmed, I can find a calm place inside.” This exclusion was supported by multiple studies, which concluded that the MAIA employs precise and effective items to ensure accurate targeting and reliability (Blackwood et al., 2023).

### Scale limitations

While invaluable in capturing subjective experiences and mental states, self-report instruments inherently carry the risk of measurement biases and distortions (Paulhus and Vazire, 2007). Issues such as reporting bias, state dependencies, social desirability, and recall bias pose significant challenges to the accuracy of self-reported data (Campbell et al., 2023). Specifically, individuals may misjudge their abilities in the context of IA, leading to overestimations or underestimations that skew the findings (Herbert and Pollatos, 2012). This variance in self-perception can be particularly pronounced across different age groups, affecting the results of IA assessments in children and the elderly (Nusser et al., 2021; Raimo et al., 2021). Moreover, the repetitive administration of self-report scales risks measuring the effects of conceptual learning rather than genuine changes in the construct of interest, as highlighted by Mehling (2016). Despite these limitations, the appeal of self-report measures lies in their simplicity, cost-effectiveness, and efficiency, requiring minimal resources beyond the participant’s willingness to engage (Paulhus and Vazire, 2007).

The MAIA scale, specifically concerning the cognitive aspects of IA, presents additional shortcomings. Its reliance on cognitive processing can disadvantage individuals with lower educational levels or language comprehension difficulties, as noted in Mehling et al.’s (2013) study. Even as the scale has been adapted to multiple languages, cultural differences in the perception and reporting of bodily sensations may further complicate the interpretation of responses.

A notable issue within the MAIA framework is the low internal consistency reported in two of its eight subscales—“Not-Distracting” and “Not-Worrying”—each comprising three items predominantly phrased in negative terms (Mehling, 2016). This construction contrasts with the scale’s otherwise positive itemization, potentially affecting the reliability scores of these subscales due to reverse scoring and the limited number of items, which influences Cronbach’s alpha—a measure sensitive to scale length (Mehling et al., 2018).

In response to these critiques, the development of MAIA-2 aimed to refine the scale, incorporate additional items into the problematic subscales, and undertake thorough psychometric evaluations. The resulting 37-item MAIA-2 demonstrates enhanced psychometric robustness, addressing prior concerns without altering the core subscales. The MAIA-2 has the potential to serve as a pivotal tool in measuring interoceptive sensibility, as evidenced by its ranking among the seven leading self-report tests for accurately capturing this construct (Todd et al., 2022). The updated scale, with new items highlighted in bold, is summarized in Table 1.

### Exploring the gender dynamics in IA

The exploration of gender differences within IA remains an emerging field of research, marked by a notable scarcity of in-depth studies (Prentice et al., 2022). Novel research indicates varied patterns in interoceptive accuracy and awareness, with women generally demonstrating lower accuracy but higher awareness scores than men (Prentice et al., 2022; Prentice and Murphy, 2022). These findings align with the broader notion that proficiency in one aspect of interoception does not necessarily predict proficiency across other interoceptive dimensions (Garfinkel et al., 2015; Pollatos and Georgiou, 2016; Ferentzi et al., 2019; Prentice and Murphy, 2022). Several factors may influence these gender disparities, including biological differences such as variances in peripheral afferent pathways and hormonal influences, physiological changes experienced through adolescence, menstrual cycle fluctuations, pregnancy, and menopause, sociocultural influences encompassing past experiences, culture, demographic context, and socialization processes (Murphy et al., 2019; Prentice et al., 2022).

Research findings suggest that females generally report more frequent perceptions of bodily sensations, deeper comprehension of the links between bodily sensations and emotional states, heightened emotional distress in response to discomfort or pain, and a tendency to view their bodies as less secure (Grabauskaitė et al., 2017). This heightened attentiveness to internal states possibly influences women’s greater likelihood of monitoring for symptoms indicative of potential health issues, interpreting sensations as warning signals, and discussing bodily sensations with others (Whitaker et al., 2015).

Specifically, within the MAIA framework, women have been found to score higher on subscales like Noticing (detection and focus on interoceptive stimuli), Body Listening (attentive insight-seeking from bodily cues), and Emotional Awareness (linking physical sensations to emotional expressions). Conversely, they tend to score lower on subscales such as Not-Worrying (disposition toward unconcerned attitudes about discomfort) and Trusting (viewing the body as safe and dependable) compared to men (Grabauskaitė et al., 2017; Luo, 2021; Re et al., 2023).

A thorough examination integrating both experimental and behavioral research is pivotal for a comprehensive understanding of gender differences in IA. The need for future studies to delve deeper into these distinctions clearly highlights an area ripe for further academic exploration and understanding.

### IA in clinical settings

There is emerging evidence for using the MAIA scale in clinical and experimental research settings. We posit that the MAIA scale harbors significant clinical utility, particularly as a screening tool that could enhance diagnostic processes and inform therapeutic approaches. Our focus on the MAIA scale is driven by its potential to elucidate aspects of body perception and emotional awareness—key domains influenced by interoceptive mechanisms. Thus, the MAIA scale emerges as a promising instrument, offering multidimensional insights into IA with considerable relevance across various clinical contexts.

Our investigation specifically targets the application of the MAIA scale within clinical populations, examining its efficacy in the context of health conditions and mental disorders such as eating disorders, stress, anxiety, depression, autism spectrum disorder, chronic pain, and suicidal ideation. To this end, we embarked on an exhaustive literature review, employing scientific databases and utilizing keywords related to interoception and the aforementioned health conditions alongside the MAIA scale. Our selection criteria were stringent, including only those studies that deployed the MAIA questionnaire to explore IA in the context of the specified clinical conditions.

### Methodological approach

To conduct this comprehensive review, we systematically accessed three major scientific databases: Scopus, PubMed, and J-Stor. Our search strategy did not impose time restrictions, aiming to encompass a broad spectrum of research. We employed key search terms aligned with our focus: MAIA, interoception, and specific conditions, including eating disorders, bulimia, anorexia, stress, anxiety, depression, autism spectrum disorder, chronic pain, and suicide ideation.

Inclusion criteria included studies published in English, Spanish, or Portuguese, studies that utilized the MAIA scale to assess interoception, and research focusing on stress, depression, eating disorders, anxiety, autism spectrum disorder, chronic pain, and suicide ideation. Studies were excluded if they did not employ the MAIA scale and did not relate to the specified topic.

Upon reviewing the abstracts based on these criteria, 18 studies met our requirements. Notably, the majority of these studies were ranked in Q1 or Q2 journals according to their Journal Impact Factor quartiles. These studies are summarized in Table 2.

**Table 2:** Studies that reported MAIA subscales*.

**Study**	***N***		**MD**	**MAIA subscales**											
		**Gender**		***N***		**ND**	**NW**	**AR**	**EA**		**SR**		**BL**		**T**
Brown et al. (2017)*	376	94% W	ED	–		X	–	–	–		X	X	X	X	X
Monteleone et al. (2021)	150	100% W	ED	–		–	–	–	–		X		X		X
Perry et al. (2021)	102	95.93% W	ED	–		–	–	X	–		X		–		X
Phillipou et al. (2022)	80	100% W	ED	NR		NR	NR	NR	NR		NR		NR		NR
Fazia et al. (2024)	90 GE 55 GC	100% W	ED	–		–	–	–	–		–		–		–
Willem et al. (2019)	165	76.4% W	O	X		–	–	–	–		–		–		X
Liné et al. (2022)**	05	100% W	O	–		–	–	–	–		X		X		–
Loucks et al. (2021)	47 GE 49 GC	67% W	Stress	NR		NR	NR	NR	NR		NR		NR		NR
Fazia et al. (2021)	30 GE 29 GC	68% W	Stress	–		–	–	–	X		X		X		X
Suzuki et al. (2021)	10,672	45.8% W	Anx	X		–	–	–	X		X		X		–
Narapareddy et al. (2022)	48 GE 68 GC	41.6% W	Anx	X		–	X	–	–		–		–		–
Vabba et al. (2023)	245	51.42% W	Anx	X		X	–	–	–		X		–		X
Edwards and Lowe (2021)	230	51% W	Anx	X		X	–	–	–		X		–		X
De Jong et al. (2016)	14 GE 26 GC	**–**	D/Stress	–		X	–	–	–	–	–		–		–
Edwards and Lowe (2021)	230	51% W	D	X		X	–	–	–		X		–		X
Vabba et al. (2023)	245	51.42% W	D	X		–	–	–	–		X		–		–
Suzuki et al. (2021)	10,672	45.8% W	D	X	X	–	–	–	X		–		X		–
Di Lernia et al. (2019)	54	81.5% W	D	X		–	–	–	X		X		X		X
Dunne et al. (2021)	281	63% W	D	X		–	–	–	–		–		X		X
Desdentado et al. (2022)	391	61% W	D	–		–	X	–	X		–		–		X
Eggart and Valdés-Stauber (2021)	87	56.32% W	MDD	–		–	X	X*	–		X*		–		X
Eggart et al. (2021)	110	55.45% W	MDD	X		–	X	–	X		–		–		–
Mul et al. (2018)	52	33.33% W	ASD	X		X	X	–	X		X		X		X
Mulcahy et al. (2019)	74 GE 20 GC	48.64% W	ASD	X		–	–	X	–		–		–		–
Yang et al. (2021)	1,360	**–**	ASD	NR		NR	NR	NR	NR		NR		NR		NR
Edwards (2022)	224	**–**	ASD	–		–	–	X	–		X		–		–
Yang et al. (2023)	62	69% W	ASD	NR		NR	NR	NR	NR		NR		NR		NR
Mehling et al. (2013)	435 GE 318 GC	53% W	CP	–		–	X	–	–		–	–	–	–	–
Di Lernia et al. (2020)	60 GE 20 GC	78.3% W	CP	–		–	–	–	–		–		–		–
Park et al. (2021)	30	53.3% W	CP	X		–	–	X	X		–		X		X
Rogers et al. (2018)	537	56.1%	SI	X		–	X	–	–		–		–		X
Duffy et al. (2018)	540	55.6% W	SI	–		–	–	–	–		–		–		X
Duffy et al. (2019)	First G 511 Second G 167	73.7% W	SI	–		–	–	–	–		–		–		X
Forkmann et al. (2019)	51 GE 44 GC	58.9% W	SI	–		–	X	–	–		–		–		–
Rogers et al. (2021)	245	56.1% W	SI	–		–	–	–	–		–		–		X
Hielscher and Zopf (2021)	Review	**–**	SI	–		–	–	–	–		–		–		X
Smith et al. (2021)	First G 136 Second G 22	71.7% W 55.7% W	SI	–		–	–	–	–		–		–		X
Gioia et al. (2022)	43	93.2% W	SI	–		–	–	–	X		–		X		X

The letter X in each column indicates that the subscale of MAIA was associated with the condition studied, e.g., the mental disorder (MD) mentioned. Colors indicate correlations between subscales found in some studies; e.g., Brown et al. (2017) showed that Attention Regulation was correlated with Self-Regulation. ED, eating disorder; O, obesity; Anx., anxiety; D, depression; MDD, major depression disorder; ASD, autism spectrum disorder; CP, chronic pain; SI, suicidal ideation; AS, attempt suicide. Subscales: N, noticing; ND, not-distracting; NP, not-worrying; RA, attention regulation; EA, emotional awareness; SR, self-regulation; BL, body listening; C, confidence. NR, not reported; N, number of people in the sample; W, women; M, men. T, disorder; X*, moderate or small effect; GE, experimental group; CG, control group. *The sample was composed of adults and adolescents. **The sample consisted of adolescent women.

## Results

### Interactions between eating disorders and interoceptive awareness: insights from the MAIA scale

Eating disorders (EDs) are characterized by profound disturbances in eating behaviors and related mental health issues, including anorexia nervosa (AN), bulimia nervosa (BN), and, notably, obesity, reflecting a broad spectrum of psychopathological concerns (Schmidt et al., 2016; Amianto et al., 2021).

Studies have identified a negative correlation between IA—particularly Emotion Regulation—and EDs, linking interoceptive deficits to elevated anxiety levels. Such deficits suggest a diminished awareness of bodily cues for emotional processing and a lack of confidence in bodily sensations essential for behavioral guidance (Brown et al., 2017; Monteleone et al., 2021). Key findings indicate significant associations between EDs and specific MAIA subscales, including Body Listening, Self-Regulation, Not-Distracting, and Trusting, suggesting that attuning to and trusting bodily sensations may be important to managing ED symptoms and recovery processes.

Further research by Monteleone et al. (2021) and Perry et al. (2021) reinforces the role of subscales such as Attention Regulation and Self-Regulation, emphasizing the difficulties in leveraging bodily sensations to regulate emotions and discomfort among individuals with ED (Monteleone et al., 2021). Phillipou et al. (2022) expanded upon these findings by integrating the “Noticing” subscale into their analysis. They proposed that specific aspects of IA, such as an increased awareness of bodily sensations and a reduced trust in one’s bodily signals, may be associated with AN symptomatology, including the neglect of hunger cues, and could represent trait factors that heighten the risk of developing AN (Phillipou et al., 2022).

A novel study by Fazia et al. (2024) investigated how depression, IA, and alexithymia mediate the relationship between ED and pain perception. The study found that individuals with EDs exhibited reduced pain sensitivity compared to healthy controls. Notably, it highlighted the role of depressive symptoms, either independently associated with alexithymia (i.e., depression is associated with an increased likelihood of perceiving greater pain) or a marked reduction in IA among those with EDs, indicating that depression may compromise IA (Fazia et al., 2024).

Studies on obesity have highlighted challenges in attending to bodily sensations, with individuals exhibiting difficulties in Noticing, Trusting, and utilizing bodily cues to inform behavioral changes (Willem et al., 2019). Liné et al. (2022) found that girls with obesity exhibited an inconsistent capacity to concentrate on their entire body without being disturbed by sensations of discomfort, a tendency exacerbated by an over-rationalization (or denial) of their perceptions. However, the authors noted a discrepancy in their study regarding the outcomes of MAIA. They suggested that these results might not accurately capture the body-self-perception verbalized by the adolescent girls interviewed. This discrepancy arises because the girls’ descriptions of their bodily sensations indicated a heightened level of interoception than what the MAIA assessments revealed (Liné et al., 2022).

### Exploring IA in stress, depression, and anxiety disorders

Research on stress has increasingly been connected to mindfulness interventions, as evidenced by studies from de Jong et al. (2016), Fazia et al. (2021), and Loucks et al. (2021). Notably, Fazia et al. (2021) conducted an experimental study on the effects of a 12-week Mindfulness-Based Stress Reduction (MBSR) program, observing a statistically significant enhancement in four interoceptive dimensions that are often impacted by stress: Emotional Awareness, Self-Regulation, Body Listening, and Trusting. These findings paralleled those of Di Lernia et al. (2019), who reported similar outcomes in their investigation of anxiety and depression. Furthermore, Loucks et al. (2021) indicated significant improvements in depressive symptoms following stress treatment, suggesting these benefits may be associated with interoceptive awareness and Self-Awareness enhancements.

Longitudinal studies (0–8 weeks) on mindfulness-based cognitive therapy (MBCT) highlighted the mediating role of the Not-Distracting subscale in reducing depressive symptoms (de Jong et al., 2016). Furthermore, the research identified a negative association between moderate to severe depression and the subscales of Noticing, Body Listening, Emotional Awareness, Self-Regulation, and Trusting (Dunne et al., 2021). Studies on Major Depressive Disorder (MDD) have consistently reported low scores on Trusting, Attention Regulation, and Not-Worrying, suggesting that interoceptive connection is impaired in depression (Eggart et al., 2021).

Suzuki et al. (2021) and Vabba et al. (2023) explored the maladaptive aspects of IA during the pandemic and reported significant associations between the Noticing subscale and maladaptive behaviors. Anxiety demonstrated positive correlations with Emotional Awareness and Body Listening, suggesting nuanced interactions between anxiety and IA.

Edwards and Lowe (2021), alongside Desdentado et al. (2022), found a significant relationship between alexithymia, depression, anxiety, and interoceptive states. These findings support models that emphasize the importance of body awareness in managing emotional processes and suggest that engaging with rather than avoiding bodily sensations may be beneficial in addressing depressive symptoms.

#### Gender differences and treatment outcomes

Research indicates gender-specific predictors of treatment response; for women, experiencing the body as safe and reliable was linked to better outcomes in depression treatment, whereas for men, the key was regulating psychological stress through physical sensations (Eggart and Valdés-Stauber, 2021).

### Interplay between autism spectrum disorder (ASD) and IA: unraveling through the MAIA scale

Sensory perception has been found to vary significantly among individuals with ASD, suggesting that interoception may be a critical component of the disorder (Williams et al., 2023). To delineate IA in ASD, Mul et al. (2018) categorized the MAIA scale into three clusters to examine ASD-related interoceptive discrepancies: “awareness” (Trusting, Emotional Awareness, Noticing), “active and reactive strategies” (Not-Distracting, Not-Worrying, Self-Regulation, Body Listening), and “attention regulation” (Attention Regulation subscale). Findings revealed that individuals with ASD displayed significantly lower scores in the “awareness” and “active and reactive strategies” clusters, with no marked differences in “attention regulation.” This suggests a pronounced IA deficit within the ASD population, excluding aspects related to attention regulation (Mul et al., 2018).

#### Correlations and causal models

Further analyses by Mul et al. (2018) and Yang et al. (2021) indicated that reduced interoceptive processing in individuals with ASD was linked to decreased self-reported IA, affecting their capacity to recognize bodily sensations and, by extension, identify and articulate emotions. These outcomes align with Edwards (2022), who explored causal relationships involving alexithymia, obsessive-compulsive disorder (OCD), and ASD, discovering significant connections with IA, notably through the subscale of Self-Regulation. Furthermore, Edwards’ study revealed that IA, particularly the Attention Regulation subscale, could partially mediate the association between OCD and ASD, suggesting indirect pathways linking alexithymia and OCD with ASD severity *via* IA (Edwards, 2022).

#### Functional connectivity and trait correlations

Yang et al. (2023) examined resting-state functional connectivity, revealing a negative correlation between MAIA scores and autistic traits, whereas Mulcahy et al. (2019) found no significant effects using the total MAIA score. Significant initial findings emerged, specifically within the Noticing and Attention Regulation subscale. This aligns with other research, such as Rogers et al. (2021), which has corroborated the advantage of analyzing the MAIA through its subscales instead of aggregating it into a single score (Rogers et al., 2021).

### Chronic pain and its intersection with IA through the MAIA scale

Chronic pain, defined as pain persisting for over 3 months or beyond the expected recovery period, often lacks a resolution due to an enduring organic cause (Di Lernia et al., 2016).

A seminal study by Mehling et al. (2013) compared primary care patients with lower back pain—categorized into recovered and unrecovered groups—with a control group of mind–body therapy practitioners. The unrecovered patients reported significantly lower scores on Not-Worrying, alongside negative correlations with pain catastrophizing and fear-avoidance behaviors. In contrast, those engaged in mind–body practices like yoga demonstrated elevated IA across all eight MAIA scales, highlighting the potential of such practices in enhancing IA (Mehling et al., 2013).

Another study by Park et al. (2021) investigated IA in patients undergoing Thoracic Spinal Cord Stimulation for pain management, noting that higher Body Listening scores were linked to reduced pain levels. Additionally, improved Attention Regulation scores were correlated with better disability outcomes and the affective dimension of pain, which might suggest that IA plays a role in mitigating the impact of pain. Contrasting findings emerged from Di Lernia et al. (2020), where no significant differences were observed across all MAIA subscales when comparing chronic pain patients (across primary, secondary, and neuropathic conditions) to healthy controls (Di Lernia et al., 2020).

### Suicide ideation (SI) and IA: insights from MAIA scale studies

The complex relationship between SI and IA has been examined in numerous studies (Duffy et al., 2018; Rogers et al., 2018, 2021; Forkmann et al., 2019; Hielscher and Zopf, 2021; Gioia et al., 2022; Smith et al., 2022), employing the MAIA scale across various contexts, including exercise dependence, depression, agitation, and pain tolerance. A recurring theme across these investigations is the role of diminished trust in bodily sensations as a critical aspect of impaired IA, influencing its connection with SI.

Research by Rogers et al. (2018) highlighted variability among individuals with a history of SI, noting distinct interoceptive difficulties: those with suicidal thoughts tended to have heightened concerns about bodily feelings, whereas individuals who attempted suicide often diverted attention from bodily sensations and struggled with using bodily cues for regulation. This dichotomy underscores two interoception challenges—redirecting attention from distressing bodily sensations among those with suicide attempts and the self-control seen in those without suicidal history (Rogers et al., 2018).

A systematic review by Hielscher and Zopf (2021) consolidated findings on the link between SI and IA, finding that a lack of body trust consistently correlates with suicidal thoughts and behaviors. Conversely, an intervention study by Smith et al. (2021) introduced an internet-based program aimed at enhancing IA, noting that improved confidence in bodily sensations was associated with decreased SI, suggesting potential therapeutic avenues. Overall, the most reliable results on the suicidal spectrum showed that body trust was linked to both thoughts of suicide and actual suicidal actions in numerous studies (Duffy et al., 2018; Rogers et al., 2018, 2021; Hielscher and Zopf, 2021; Gioia et al., 2022; Smith et al., 2022).

Additionally, examining the gender composition within the study samples previously mentioned revealed a predominance of female participants, with mixed-methods studies occasionally overlooking gender-based analysis. Nonetheless, specific investigations (e.g., Willem et al., 2019; Di Lernia et al., 2020; Dunne et al., 2021; Edwards, 2022) into gender differences have confirmed that women have an enhanced ability to recognize emotions and bodily sensations. Table 2 summarizes the results described above according to MAIA subscales.

## Discussion

The importance of bodily symptoms and their perception has long been recognized in psychopathology (Kapfhammer, 2006). Despite the established significance of IA, its systematic application in clinical screening and diagnostic support remains underutilized, finding broader applications in clinical research and experimental studies. The MAIA scale may serve as a tool in these endeavors by better characterizing clinical populations.

The reviewed literature suggests that IA deficits may be common in the development and perpetuation of various mental health issues, including eating disorders (ED), stress, suicide ideation (SI), depression, and chronic pain. Specifically, avoidance of uncomfortable bodily sensations and distrust in bodily signals appear to exacerbate symptomatology across these conditions (Mehling et al., 2013; Schulz and Vögele, 2015; Brewer et al., 2016; Brown et al., 2017; Duffy et al., 2018; Khalsa et al., 2018; Rogers et al., 2018, 2021; Forkmann et al., 2019; Dunne et al., 2021; Hielscher and Zopf, 2021; Monteleone et al., 2021; Park et al., 2021; Smith et al., 2021; Gioia et al., 2022; Phillipou et al., 2022). Conversely, enhanced IA may be indicative of both physical and psychological wellbeing.

### Homeostatic dysregulation and interoceptive deficits

The interconnection between certain mental and somatic disorders can be attributed to homeostatic dysregulation and deficits in IA, as suggested by Furman et al. (2013), Khalsa et al. (2018), Dunne et al. (2021), Eggart et al. (2021), Eggart and Valdés-Stauber (2021), and Park et al. (2021). These disorders are further characterized by a reduced awareness and trust in bodily signals, leading to a limited or complete absence of the use of bodily information (Bechara et al., 2000; Furman et al., 2013; Hielscher and Zopf, 2021). A lack of trust in bodily sensations is interpreted as difficulty maintaining and regulating attention to bodily signals. Moreover, challenges in accurately recognizing and labeling one’s emotional and physical states have been identified as critical indicators of a higher risk for suicidal thoughts, intentions, and actions (Hielscher and Zopf, 2021). Research also indicates that IA is significant in the development and persistence of state and trait anxiety, anxiety sensibility, and anxiety disorders (Domschke et al., 2010). This influence may relate to aspects of Emotional Awareness, particularly in conditions of hyperarousal, as well as to Self-Regulation and Body Listening, which are also affected by hyperarousal scenarios.

### IA and autism spectrum disorder

The “interoception-alexithymia-autism” theory (Quattrocki and Friston, 2014; Tang et al., 2020; Edwards, 2022) posits that irregular IA may be a precursor to alexithymia, potentially heightening the risk for ASD. Studies examining this theory show a link between IA and autistic traits, alexithymia, and empathy (Mul et al., 2018; Yang et al., 2022). Edwards (2022) further integrate these findings into broader theories of interoception as predictive coding errors (Seth, 2013; Barrett and Simmons, 2015; Barrett et al., 2016; Seth and Friston, 2016; Stephan et al., 2016; Owens et al., 2018). This interpretation suggests mindfulness and emotion recognition practices could mitigate autistic conditions by reducing interoceptive prediction errors.

## Conclusion

The research reviewed here suggests an association between interoceptive awareness and various mental health conditions, as quantified by the MAIA scale. Lower scores on this scale often indicate maladaptive behaviors and diminished IA, linked to various health conditions. Notably, engaging in higher-order interoceptive processes—such as enhanced body awareness, self-regulating through bodily cues, and fostering trust in one’s bodily signals—correlates with improved subjective wellbeing and may offer resilience against the deleterious effects of stress and other adverse life experiences.

### Implications for clinical practice and research

These findings advocate for a greater integration of IA assessments in clinical settings, not only as a diagnostic aid but also as a component of therapeutic strategies. Engaging patients in practices that enhance IA could offer a promising avenue for addressing psychopathological conditions and healthcare and providing valuable insights into patients’ abilities to perceive and interpret bodily sensations (Khalsa et al., 2018).

### Acknowledging limitations

A considerable portion of the studies employing the MAIA scale have been correlational, highlighting the need for more experimental research incorporating this instrument. This approach would more definitively elucidate causal relationships between IA and mental health conditions. Additionally, while some studies did not primarily focus on stress, depression, or anxiety, these conditions emerged as significant predictors or co-occurring issues, suggesting broader applications of IA assessments in diverse mental health contexts.

### Future research directions

Expanding the use of the MAIA scale into clinical populations and those from vulnerable backgrounds, including individuals impacted by violence and trauma, alongside more focused research on adolescents and children, presents a promising avenue for future investigations. Such expansion would diversify the populations studied and enhance our understanding of interoceptive awareness across different life stages and experiences.

Longitudinal studies employing the MAIA scale could significantly contribute to unraveling the temporal dynamics between interoceptive awareness and the development or progression of psychopathological conditions. By exploring these temporal relationships, researchers could better determine whether alterations in interoceptive awareness precede the onset of mental health issues or arise as a result of existing psychopathological symptoms.

Furthermore, given the predominance of female participants in existing studies, future research should aim to systematically explore age and gender differences in interoceptive awareness. Such studies would offer valuable insights into how interoception may manifest differently across genders and developmental stages. Exploring mind–body interventions for interoceptive awareness holds the potential for therapeutic advancements which may mitigate the impact of psychopathological symptoms.

## Author contributions

PS, J-PM, and DH conceived and designed the study. PS and J-PM contributed analysis and interpretation. All authors participated in drafting the manuscript, contributed to the study, and approved the submitted version.

## Footnotes

^1^ https://osher.ucsf.edu/research/maia
